# Specific Expression of Antimicrobial Peptides from the Black Soldier Fly in the Midgut of Silkworms (*Bombyx mori*) Regulates Silkworm Immunity

**DOI:** 10.3390/insects14050443

**Published:** 2023-05-08

**Authors:** Xuan Deng, Lianlian Liu, Jing Deng, Xingfu Zha

**Affiliations:** State Key Laboratory of Resource Insects, College of Sericulture, Textile and Biomass Sciences, Southwest University, Chongqing 400715, China

**Keywords:** transgenic silkworm, midgut, black soldier fly, antimicrobial peptide, transcriptome sequencing

## Abstract

**Simple Summary:**

Antimicrobial peptides (AMPs) are major components of the insect innate immune system and are involved in multiple antimicrobial and antiviral responses. The black soldier fly is an insect that has received substantial attention in recent years; however, few functional studies on its antimicrobial peptides have been performed. In this study, we specifically induced the expression of the antimicrobial peptide genes *HiCG13551* and *Hidiptericin-1* of the black soldier fly in the midgut of silkworms. *Hidiptericin-1* expression in the midgut of silkworms helped to enhance its immune response and imparted superior resistance against *Staphylococcus aureus* infection, while *HiCG13551* expression in silkworms tended to weaken the antimicrobial effect. In addition, we performed transcriptome sequencing of midgut tissues after *S. aureus* infection to explore the expression of immune-related genes in the overexpressed strain and found that endogenous antimicrobial peptides, reactive-oxygen-species-related genes, pattern recognition receptors, and immunomodulatory factors were up-regulated in the silkworms due to the transgenic overexpression of *Hidiptericin-1*. All these results indicated that the ov-AMP49 had better antibacterial activity.

**Abstract:**

Antimicrobial peptides are molecules with strong antimicrobial activity and are of substantial interest for the immunization of insects. As a type of dipteran insect that can turn organic waste into animal feed, the black soldier fly (BSF) can “turn waste into treasure”. In this study, we investigated the antimicrobial activity of the antimicrobial peptide genes, *HiCG13551* and *Hidiptericin-1,* of BSF in silkworms, by overexpressing the genes specifically in the midgut. Changes in the mRNA levels of the transgenic silkworms after infection with *Staphylococcus aureus* were evaluated using transcriptome sequencing. The results showed that *Hidiptericin-1* had stronger antimicrobial activity than *HiCG13551*. KEGG enrichment analysis showed that the differentially expressed genes in the transgenic overexpressed *Hidiptericin-1* silkworm lines from the D9L strain were mainly enriched in the starch and sucrose metabolism, pantothenate and CoA biosynthesis, drug metabolism (other enzymes), biotin metabolism, platinum drug resistance, galactose metabolism, and pancreatic secretion pathways. In addition, immune-related genes were up-regulated in this transgenic silkworm strain. Our study may provide new insights for future immune studies on insects.

## 1. Introduction

The silkworm is an important model insect and is sensitive to multiple Gram-negative and Gram-positive bacteria and viruses [1]. Antimicrobial peptides (AMPs) have a range of antibacterial, antifungal, and antiviral activities [2]. It has been reported that the antimicrobial peptides in the silkworm mainly include Cecropins, Attacins, Lebocins, Gloverins, Moricins, and Defensins [3]. Furthermore, it has been reported that the AMP family of silkworm has better antimicrobial activity than those of *Drosophila* [3].

The black soldier fly (BSF), *Hermetia illucens* (L.) (Diptera: Stratiomyidae), can effectively exploit organic wastes that can be used as feed for many aquaculture species and poultry [4]. Since the larvae of BSF inhabit humid and dark environments that are likely to harbor bacteria, scholars have hypothesized that the species’ immune system is well developed and contains a variety of antimicrobial peptide genes [5]. In 2020, a number of genes involved in the immune system and digestive system were analyzed in a high-quality BSF genome. Among them, 50 antimicrobial peptide genes were observed, a total which is much larger than the number of antimicrobial peptide genes in the silkworm itself [5,6]. To verify that the expression of antimicrobial peptide genes from BSF in the silkworm can enhance its immunity, a transgenic silkworm strain combining three antimicrobial peptide genes from BSF was obtained and found to have enhanced resistance to both Gram-positive and Gram-negative bacteria [7].

The midgut of the silkworm plays important roles in aiding digestion and defending against pathogens. The midgut is the first line of defense against invading pathogens; epithelial cells produce reactive oxygen species (ROS) and AMPs to protect the host from pathogenic microorganisms and ensure homeostasis of the symbiotic microbial community and normal gut functioning [8,9,10]. In 2015, the P3P + 5 UI promoter was obtained via cloning in the midgut of the silkworm and proved to be a midgut-specific promoter [11].

In this study, we used the P3P + 5UI promoter to specifically overexpress antimicrobial peptide genes from BSF in the silkworm midgut in order to investigate immunity in silkworms. Transgenic silkworm strains with strong antibacterial activities were obtained. The common silkworm and transgenic silkworm were infected with *Staphylococcus aureus* and *Escherichia coli*, and it was found that *S. aureus* stimulated a stronger immune response. Transcriptome sequencing analyses were then performed on midgut tissues taken from the two strains of silkworm 12 hours after infection with *S. aureus* to investigate the effects of the exogenous genes *HiCG13551* and *Hidiptericin-1* on the immune system of the silkworms on the RNA level. KEGG enrichment analysis showed that the differentially expressed genes in the transgenic overexpressed *HiCG13551* silkworm lines were mainly enriched in the pancreatic secretion, starch and sucrose metabolism, and protein digestion and absorption pathways, while the differentially expressed genes in the transgenic overexpressed *Hidiptericin-1* silkworm lines were mainly enriched in the starch and sucrose metabolism, pantothenate and CoA biosynthesis, drug metabolism (other enzymes), biotin metabolism, platinum drug resistance, galactose metabolism, and pancreatic secretion pathways.

## 2. Materials and Methods

### 2.1. Black Soldier Fly and Silkworm Strains

Black soldier fly (*Hermetia illucens*) eggs were purchased from Nanjing Hei Shui Meng (Nanjing, Jiangsu, China). The rearing methods and environmental conditions followed those used in a previous study [12]. The D9L silkworm strain was provided by the State Key Laboratory of Silkworm Genome Biology (Southwest University, Chongqing, China).

### 2.2. RNA Extraction and cDNA Synthesis 

Total RNA was extracted using TRIzol reagent (Invitrogen, Carlsbad, CA, USA), and the concentration and purity of the RNA samples were verified using a spectrophotometer. First-strand cDNA was synthesized via reverse transcription of RNA using the M-MLV Reverse Transcriptase (M1701, Promega, Madison, WI, USA).

### 2.3. Cloning of the AMP Genes 

Two BSF genes, *HiOGS03714* and *HiOGS01149*, have been shown to contribute to the antimicrobial activity of silkworms. These two genes were annotated as CG13551 and diptericin, respectively [7]. Therefore, we subsequently renamed *HiOGS03714* and *HiOGS01149* as *HiCG13551* and *Hidiptericin-1,* respectively.

*HiCG13551* and *Hidiptericin-1* were cloned via PCR using TransTaq^®^ High Fidelity (HiFi) DNA Polymerase (TransGen Biotech, Beijing, China). The cycling parameters were as follows: 94 °C for 4 min, followed by 30 cycles of 94 °C for 40 s, 55 °C at the appropriate annealing temperature for 40 s, and 72 °C for 30 s. The PCR products were recycled and cloned into the PEASY-T5 ZERO vector. Two positive clones were selected for each gene and sequenced by the Beijing Genomics institution (BGI, Beijing, China). 

### 2.4. Recombinant Vector Construction and Transgenic Silkworm Generation

*HiCG13551* and *Hidiptericin-1* ORF PCR products with a length of 258 bp and 546 bp, respectively, were cloned into the pEASY-T5 Zero vector. The constructed plasmids were named *HiCG13551*-T5 and *Hidiptericin-1*-T5, respectively. P3P + 5UI is a specific promoter with activity throughout the entire silkworm midgut [11]. To verify the regulatory roles of *HiCG13551* and *Hidiptericin-1* genes in midgut immunity in silkworms, we combined the *HiCG13551*-T5 and *Hidiptericin-1*-T5 plasmids with pSL1180-P3P + 5UI after digestion with NotI and BamHI and named the resulting vectors as pSL1180 [P3P + 5UI-HiCG13551-SV40] and pSL1180 [P3P + 5UI-Hidiptericin-1-SV40], respectively. Finally, the target gene was ligated with P3P + 5UI and inserted into the transgenic vector piggyBac [13]. The transgenic silkworm vectors, *piggyBac*-HiCG13551 [3 × P3-Red-SV40, P3P + 5UI-HiCG13551-SV40] and *piggyBac*-Hidiptericin-1 [3 × P3-Red-SV40, P3P + 5UI-Hidiptericin-1-SV40], were successfully constructed to obtain transgenic silkworms.

The constructed transgenic vector was mixed in equal proportions with pHA3PIG helper plasmids maintained in our laboratory, and freshly laid D9L embryos were injected using a microinjector. The injected silkworms were referred to as the G0 generation, and after normal hatching, they continued to feed the next generation, G1. When the eggs of the G1 generation had developed up to the seventh day, they were screened using a fluorescent microscope (OLYMPUS, Tokyo, Japan) under red wavelength excitation. Samples with red light reflected from the eyes of the silkworm embryos were regarded as positive individuals and were maintained until the moth stage and screened again to confirm the red light. The G1 generation moths were mated with wild-type moths, forming the G2 generation. On approximately day 7, the G2 generation embryos were screened for red fluorescence, and positive individuals were reared to the fifth larval instar for three days and dissected for subsequent experiments. The transgenic overexpressed *HiCG13551* silkworm line was named ov-AMP14, and the transgenic overexpressed *Hidiptericin-1* silkworm line was named ov-AMP49.

### 2.5. Quantitative Real-Time PCR (qRT-PCR) Analysis

In order to detect the expression of exogenous antimicrobial peptide genes in the transgenic strains, we conducted real-time fluorescence quantitative PCR to detect the relative expression levels of *HiCG13551* and *Hidiptericin-1*. According to the manufacturer’s instructions (Total RNA Kit., OMEGA, Biel, Switzerland), we extracted total RNA from each tissue of the day 3 fifth-instar silkworms, including the midgut, head, epidermis, testis, ovary, fat body, silk gland, and Malpighian tubule. The experiment was performed in three biological and technical replicates. The transgenic silkworm was used as the experimental group, and the wild-type (WT) silkworm was used as the control group.

First-strand cDNA was synthesized via reverse transcription using the Primescipt^TM^ RT reagent kit with gDNA Eraser (TaKaRa, Shiga, Japan). The specific primers for qPCR are shown in Supplementary Table S1. The reference gene was *BmSW* (GenBank accession number: XM_028181535.1). The synthesized cDNA was diluted to 200 ng/μl as the qPCR template, and quantitative PCR was performed using NovoStart® SYBR qPCR Super Mix Plus (Novoprotein, Shanghai, China). The qPCR experiments were carried out using an ABI7500 Real-Time PCR machine (Applied Biosystems, Foster City, CA, USA). All procedures with instruments and kits were performed according to the manufacturers’ instructions and protocols. The PCR mixture (10.0 µL 2 × SYBR Green Realtime Master Mix, 0.8 µL qPCR forward primer, 0.8 µL qPCR reverse primer, 6.4 µL ultrapure H_2_O, and 2.0 µL Template) was added to the qPCR reaction plate. The cycling parameters were as follows: 95 °C for 30 s, followed by 30 cycles of 95 °C for 3 s and 60 °C for 30 s. The qPCR experiment was repeated more than three times. After the reaction, we exported the data from the ABI7500 Real-Time PCR machine and analyzed these data using the 2^−ΔΔCT^ method.

### 2.6. Pathogenicity Assays of Transgenic Silkworms

*Staphylococcus aureus* and *Escherichia coli* were maintained in our laboratory. To verify that the antimicrobial peptide genes of BSF were able to enhance the immunity of silkworms after overexpression in the midgut, *S. aureus* and *E. coli* were injected into the newly molted fifth-instar larvae at a concentration of 1 × 10^8^ CFU. The bacterial concentration was determined with reference to the reported methods [14]. Each time 15 silkworms were treated, three groups of replicates were set up. The wild-type (WT) was used as the control group, and silkworms injected with PBS were regarded as the non-infectious treatment group. The three treatment groups were used to calculate the daily mortality of the larvae, and the data were analyzed using GraphPad Prism 9.5.1. The experiment was repeated again to detect the expression of endogenous antimicrobial peptides and important immune factors in the silkworms after infection with pathogenic bacteria.

### 2.7. Transcriptome Sequencing Analysis

Twelve hours after the ov-AMP14 and ov-AMP49 strains were infected with *S. aureus*, their midgut tissues were sampled. The wild type was used as the control, and we took three replicates of the midgut tissue from three individuals as one sample, for a total of twelve samples. The samples were sequenced and analyzed by Majorbio (Shanghai, China) using the Illumina Novaseq 6000 platform.

The RNA-seq transcriptome library was prepared using a TruSeqTM RNA sample preparation Kit from Illumina (San Diego, CA, USA) with 1 μg of total RNA. The synthesized cDNA was subjected to end-repair, phosphorylation, and ‘A’ base addition according to the Illumina library construction protocol. The libraries were size-selected for cDNA target fragments of 300 bp on 2% Low Range Ultra Agarose, followed by PCR amplification using Phusion DNA polymerase (NEB) for 15 PCR cycles. After quantification with TBS380, the paired-end RNA-seq sequencing library was sequenced using an Illumina HiSeq xten/NovaSeq 6000 sequencer (2 × 150 bp read length).

The raw sequencing data were submitted to the NCBI SRA database with the following accession numbers: PRJNA839509 and PRJNA839217.

### 2.8. Differential Expression Analysis and Functional Enrichment

To identify the DEGs (differential expression genes) between two different samples, the expression level of each transcript was calculated according to the transcripts per million reads (TPM) method.

RSEM (http://deweylab.biostat.wisc.edu/rsem/ (accessed on 10 February 2022)) [15] was used to calculate the gene expression abundance. Differential expression analysis was performed using the DESeq2 [16]/DEGseq [17] Q value ≤ 0.05. DEGs with a |log_2_FC| > 1 and Q value ≤ 0.05(DESeq2)/Q value ≤ 0.001(DEGseq) were considered to be significantly differentially expressed genes. GO functional enrichment and KEGG pathway analyses were carried out using Goatools (https://github.com/tanghaibao/Goatools (accessed on 15 February 2022)) and KOBAS (http://kobas.cbi.pku.edu.cn/home.do (accessed on 26 February 2022)) [18].

### 2.9. Data Analysis

Data analysis and mapping were performed with the GraphPad Prism 9.5.1. All data are expressed as the mean ± standard deviation. An unpaired two-tailed Student’s *t*-test was used to determine statistical significance. *p* < 0.05 was considered to indicate a significant difference (* *p* < 0.05, ** *p* < 0.01, and *** *p* < 0.001).

## 3. Results

### 3.1. Construction of Recombinant Vectors and Acquisition of Transgenic Silkworms

**Figure 1:**
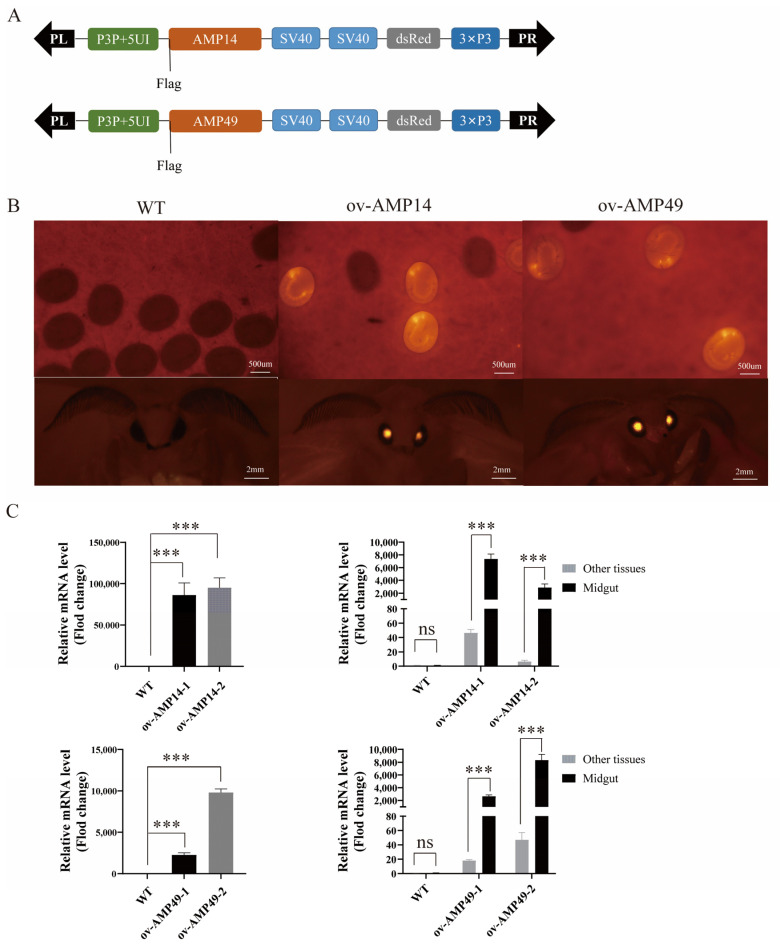
Transgenic silkworm construction. (**A**) Schematic diagram of the construction of overexpression vectors for two AMP genes. DsRed: reporter marker. SV40: polyadenylation signal. P3P + 5UI: midgut-specific promoter. (**B**) Fluorescent images of transgenic silkworms in the embryonic and moth stages. (**C**) The left panel shows the relative mRNA levels of *HiCG13551* and *Hidiptericin-1* in the midgut in overexpressing individuals and wild-type individuals, which were investigated via qRT-PCR. The graph on the right represents the relative mRNA levels of *HiCG13551* and *Hidiptericin-1* in the midgut and other tissues from the overexpression silkworms. All data are shown as the mean ± s.e.m. of three independent experiments. The expression of antimicrobial peptide genes in the midgut of the wild-type silkworms was normalized. *p*-values were determined using Student’s *t* test. *** *p* < 0.001. Other tissues represent mixing of the head, epidermis, testis, ovary, fat body, silk gland, and Malpighian tubule. ov-AMP14-1 and ov-AMP14-2 represent two transgenic lines that overexpress the *HiCG13551* gene; ov-AMP49-1 and ov-AMP49-2 represent two transgenic lines that overexpress the *Hidiptericin-1* gene.

The constructed transgenic vectors, *piggyBac*-HiCG13551 [3 × P3-Red-SV40, P3P + 5UI-HiCG13551-SV40], were injected into eggs in equal proportions with the helper plasmid (Figure 1A). Of the injected eggs, 180 (33.3%) hatched, and 146 (24.3%) survived to the adult stage. In addition, the transgenic vectors, *piggyBac*-Hidiptericin-1 [3 × P3-Red-SV40, P3P + 5UI-Hidiptericin-1-SV40], were injected into eggs, of which 198 (30.9%) hatched, and 164 (25.6%) survived to the adult stage (Table 1).

The G0 moths were self-crossed and produced the G1 generation. Positive individuals were screened using a fluorescent microscope, and those with red fluorescence in the eyes, compared to the control group, were considered positive (Figure 1B).

The two transgenic strains were named ov-AMP14 and ov-AMP49, respectively, and the expression of the *HiCG13551* and *Hidiptericin-1* genes was detected through fluorescence quantitative PCR. The results showed that both *HiCG13551* and *Hidiptericin-1* were significantly up-regulated in the midgut tissue of the transgenic silkworms compared to the control silkworms. To verify that the heterologous antimicrobial peptide genes in the ov-AMP14 and ov-AMP49 strains were expressed only in the midgut tissues, fluorescent quantitative PCR was performed on the midgut and all other tissues. The results showed that the *HiCG13551* and *Hidiptericin-1* genes were only expressed in high amounts in the midgut tissues (Figure 1C). The above results indicated that we successfully obtained transgenic silkworm lines with specific overexpression in midgut tissues.

### 3.2. Pathogenicity Assays of AMPs

**Figure 2:**
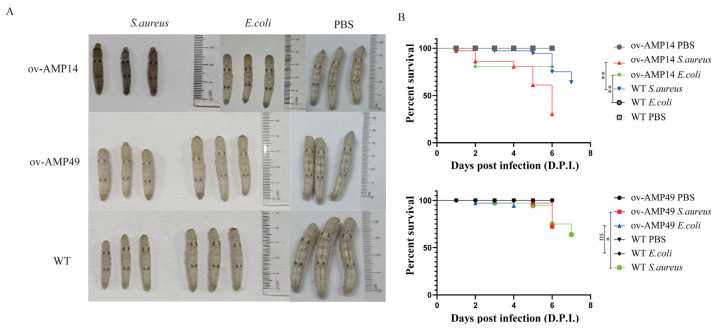
Pathogenicity assays of the AMP-overexpressing transgenic silkworms. (**A**) Phenotypic observations of the wild-type and overexpression strains infected with *S. aureus*. (**B**) Survival statistics after injection of *S. aureus* into the wild-type and overexpressing silkworms. *p*-values were determined using the log-rank test. ns means not significant; * *p* < 0.05, ** *p* < 0.01.

To test the effect of the antimicrobial peptide genes of BSF on the regulation of silkworm immunity after expression in the midgut, we conducted pathogenicity assays using the newly molted fifth instar larvae. After feeding the injected silkworms with mulberry leaves, the survival rate of the silkworms was calculated daily until the silkworms started to spin silk. We found that the transgenic silkworm strains showed a different health status from the wild-type silkworm. Among them, the transgenic strain ov-AMP49 showed no significant differences in appearance after infection with these two pathogenic bacteria, while the ov-AMP14 showed strong individual differences. We found that the experimental and control silkworms exhibited loss of appetite, slow movement, thinness, and epidermal blackening in appearance after infection with *S. aureus*. These lesions were most pronounced in the transgenic strain ov-AMP14, followed by the wild-type silkworm, and least pronounced in the transgenic strain ov-AMP49. In contrast, these silkworms exhibited only a yellowing of the tail after *E. coli* infection (Figure 2A). These results indicated that *S. aureus* exhibited greater pathogenicity relative to *E. coli* and that the transgenic strain ov-AMP49 exhibited greater antimicrobial activity.

After infection with *S. aureus*, the wild-type silkworms started to die at 3 d.p.i, and the transgenic strains ov-AMP14 and ov-AMP49 started to die at 1 d.p.i and 5 d.p.i after injection, respectively. After infection with *E. coli*, the wild-type silkworms did not die until they began to spin silk, while both the transgenic strains ov-AMP14 and ov-AMP49 started to die at 2 d.p.i. In addition, we found that the final survival rates after infection with *S. aureus* and *E. coli* were 75% and 94.5% for the transgenic strain ov-AMP49 and 30.5% and 80.5% for ov-AMP14, respectively. It was shown that the transgenic strain ov-AMP49 displayed higher survival rates (Figure 2B). Notably, upon infection with both pathogenic bacteria, *S. aureus* showed stronger pathogenic effects on the transgenic strains. the log-rank test showed that there were significant differences in both the survival time prolongation and survival rate enhancement. These results are consistent with our observation of the ov-AMP49 phenotype after infection with the pathogenic bacteria (Figure 2A).

Those data indicated that *S. aureus* caused higher mortality among the silkworms than *E. coli*, and ov-AMP49 had stronger antibacterial activity.

It has been reported that there are six different types of antimicrobial peptides in silkworms. Among them, Cecropins have activity against both Gram-negative and Gram-positive bacteria, and *Cecropin* is an antimicrobial peptide with a stable structure [19]. Gloverins are found only in Lepidoptera [20], such as silkworms (*B. mori*) [21,22]. Therefore, in this study, the expressions of *Cecropin* and *Gloverin* in silkworms were selected as indicators to detect the endogenous antibacterial peptides in the silkworm after infection with *S. aureus.* The IMD (immune deficiency) signaling pathway is an important signal transduction pathway regulating the humoral immune response of silkworms [23]. Therefore, we also detected the expression of *imd* in this study.

**Figure 3:**
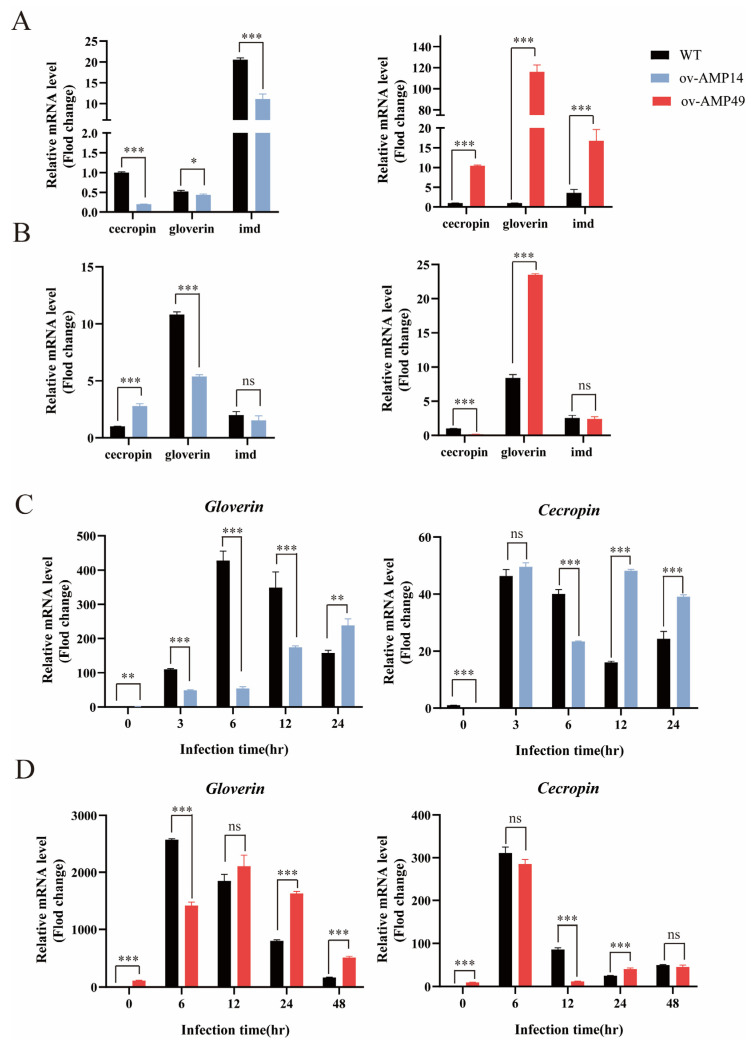
qPCR analysis of the expression of endogenous antimicrobial peptides and immune-related genes in transgenic silkworms. (**A**,**B**) Relative expression of immune-related genes in the uninfected and *S. aureus*-infected groups. The relative expression of *Cecropin* in the midgut of the wild-type silkworms was normalized. (**C**,**D**) Expression trends of endogenous antimicrobial peptides in two transgenic silkworms after 3, 6, 12, and 24 h of infection with *S. aureus*. The relative expression of *Cecropin* or *Gloverin* was normalized to that of the wild-type silkworms in the uninfected group. Black bars represent WT, red bars represent ov-AMP49, and blue bars represent ov-AMP14. All data are shown as the mean ± s.e.m. of three independent experiments. *p*-values were determined using Student’s *t*-test. * *p* < 0.05, ** *p* <0.01, *** *p* < 0.001. ns means no significant difference.

The results showed that the expression of *Gloverin, Cecropin* and *imd* in the transgenic line ov-AMP14 was significantly lower than that in the WT in the uninfected group, while the expression of the *Gloverin, Cecropin,* and *imd* in ov-AMP49 was significantly higher than that in the WT (Figure 3A). In addition, after infection with *S. aureus*, *Cecropin* was up-regulated in ov-AMP14 and down-regulated in ov-AMP49, while *Gloverin* was down-regulated in ov-AMP14 and up-regulated in ov-AMP49. The expression of *imd* was not significantly different in between ov-AMP14 and ov-AMP49 (Figure 3B).

The results showed that the expression of *Cecropin* in the transgenic line ov-AMP14 was significantly down-regulated after 6 h of injection and up-regulated after 12–24 h, while *Gloverin* expression was significantly down-regulated after 3–12 h of injection and up-regulated after 24 h (Figure 3C). After treatment with *S. aureus*, the expression of *Cecropin* in the transgenic line ov-AMP49 was significantly down-regulated 12 h after injection, up-regulated after 24 h, and equal to wild-type after 48 h, whereas the expression of *Gloverin* was significantly down-regulated 6 h after injection, equal to wild-type after 12 h, and up-regulated after 24–48 h (Figure 3D). These results suggest that *Hidiptericin-1* may enhance the expression of endogenous antimicrobial peptides and immune-related genes in silkworms, while *HiCG13551* may weaken immunity and immune-related genes.

### 3.3. Transcriptome Sequencing and Identification of Differentially Expressed Genes

**Figure 4:**
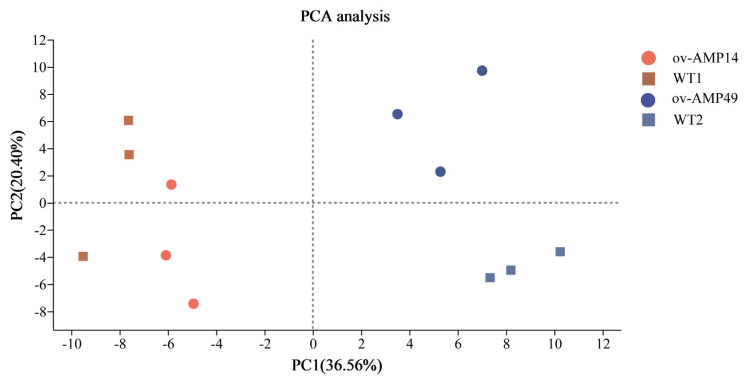
Principal component analysis (PCA) of all RNA-Seq samples. The samples of ov-AMP49 are represented by the blue triangles, and the samples of the ov-AMP14 are represented by the red dots. The samples of WT1 are represented by the green diamond pattern, and the samples of WT2 are represented by the gray square. The distance between each sample point represents the distance of the sample. Closer distances indicate a higher level of similarity between samples.

PCA analysis shows that the contribution of principal component 1 (PC1) to the sample in the two-dimensional plot was 36.56%, and the contribution of principal component 2 (PC2) to the sample in the two-dimensional plot was 20.40% (Figure 4). Therefore, these results indicate that the repeatability of the same silkworm strain is good, and an independent population was formed among the different strains.

To better understand the changes in gene expression between the wild-type and the transgenic silkworms after infection with *S. aureus*, we analyzed the differentially expressed genes (DEGs). A total of 288 DEGs were found in the midgut of the WT2 vs. ov-AMP49 group, of which 111 genes were down-regulated and 177 genes were up-regulated (Figure S1C). Transcriptome analyses of the WT1 vs. ov-AMP14 group identified 476 DEGs, of which 208 genes were up-regulated, and 268 genes were down-regulated. As shown in Figure S1, there were 213 genes specific to the WT2 vs. ov-AMP49 group and 401 genes specific to the WT1 vs. ov-AMP14 group, with 75 genes shared between the two groups. All DEGs of the experimental and control groups were clustered into one category.

### 3.4. Functional Classification by GO Enrichment Analysis

**Figure 5:**
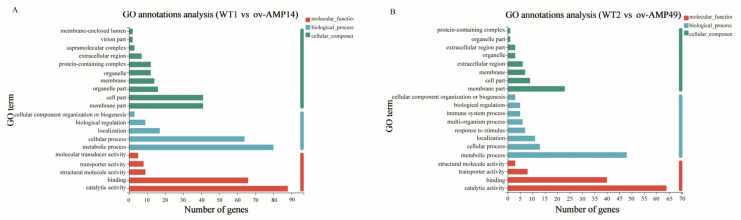
Go enrichment analysis of DEGs: (**A**) Gene ontology annotation of DEGs in WT1 vs. ov-AMP14 samples. (**B**) Gene ontology annotation of DEGs in WT2 vs. ov-AMP49 samples.

The results of the GO enrichment analyses showed that the functions of the DEGs were divided into three categories, including biological process (BP), cellular component (CC), and molecular function (MF) categories. As shown in Figure 5A, there were five, ten, and five GO terms with the most abundant DEGs in the BP (175 DEGs), CC (150 DEGs), and MF (180 DEGs) categories, respectively, in the WT1 vs. ov-AMP14 group. Moreover, the top GO terms in the MF category were “catalytic activity” (88 DEGs); in the CC category, they were “cell part” (41 DEGs) and “membrane part” (41 DEGs); and, in the BP category, they were “metabolic process” (80 DEGs).

As shown in Figure 5B, 98, 53, and 116 DEGs were distributed in the BP (eight GO terms), CC (eight GO terms), and MF (five GO terms) categories, respectively, in the WT2 vs. ov-AMP49 group. The top three GO terms in the BP, CC, and MF categories were similar to those found in the WT1 vs. ov-AMP14 group.

### 3.5. Functional Classification by KEGG Analysis

**Figure 6:**
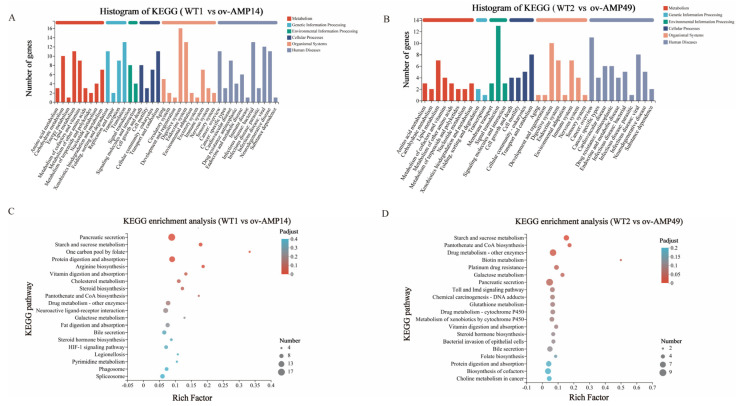
KEGG classification analysis of DEGs. (**A**) KEGG classification analysis of DEGs in WT1 vs. ov-AMP14 samples. (**B**) KEGG classification analysis of DEGs in WT2 vs. ov-AMP49 samples. (**C**) KEGG enrichment analysis of DEGs in WT1 vs. ov-AMP14 samples. (**D**) KEGG enrichment analysis of DEGs in WT2 vs. ov-AMP49 samples.

As shown in Figure 6A, 40 KEGG pathways were assigned and divided into six categories, including metabolism (50 DEGs), genetic information processing (35 DEGs), environmental information processing (12 DEGs), cell processes (29 DEGs), and organic systems (52 DEGs). The six categories contain 9, 4, 2, 4, 10, and 11 metabolic pathways, respectively. The KEGG enrichment analysis showed that the WT1 vs. ov-AMP14 group was mainly enriched in the pancreatic secretion, starch and sucrose metabolism, protein digestion and absorption, and other pathways (Figure 6C).

As shown in Figure 6B, 35 KEGG pathways were assigned and divided into six categories, among which, eight pathways belonged to metabolism (26 DEGs), two pathways were related to genetic information processing (three DEGs), three pathways were connected to environmental information processing (19 DEGs), four pathways associated with cellular processes (21 DEGs), and eight pathways were related to organic systems (32 DEGs). The KEGG enrichment analysis showed that the WT2 vs. ov-AMP49 group was mainly enriched in the starch and sucrose metabolism, pantothenate and CoA biosynthesis, drug metabolism (other enzymes), biotin metabolism, platinum drug resistance, galactose metabolism, pancreatic secretion, and other pathways (Figure 6D).

### 3.6. Analysis of the Expression of Immunity-Related Genes in the Silkworms Following Bacterial Infection

**Figure 7:**
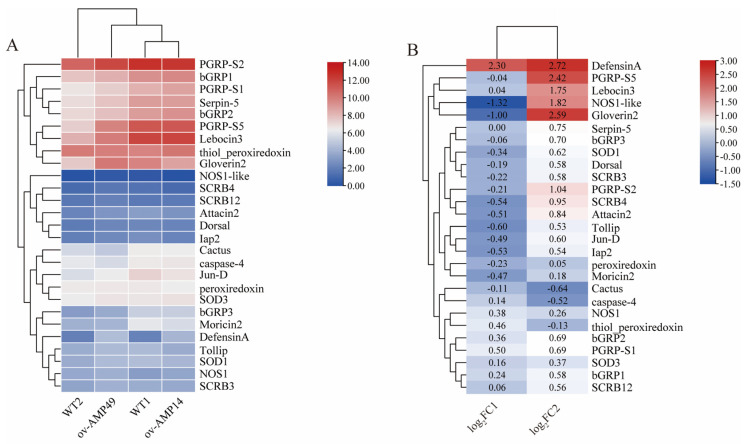
Expression analysis of immune-related genes in the midgut of silkworms. (**A**) Heat map of expression of immune-related genes in silkworms. The FPKM values of immune-related genes were taken as logarithmic values with a base of 2 to plot the expression heat map. (**B**) Expression trends of immune-related genes in silkworms. In the figure, log2FC1 represents the log2(FPKM-ov-AMP14/FPKM-WT1) value; log2FC2 represents the log2(FPKM-ov-AMP49/FPKM-WT2) value. This indicates the trend of expression of these immune-related genes in the two groups of samples.

To identify the expression of intestinal immunity-related genes in the transgenic and wild-type silkworms under bacterial infection conditions, an expression heat map was drawn using TBtools (Figure 7). The results were as follows:

The expression of endogenous antimicrobial peptides was increased in ov-AMP49, among which *DefensinA*, *Lebocin3*, and *Gloverin2* exhibited significant differences (log2(foldchange) > 1). In contrast, down-regulated expressions of *Moricin2, Attacin2*, and *Gloverin2* were observed in ov-AMP14, while only *DefensinA* was significantly up-regulated (Figure 7B). In the transgenic strain, ov-AMP49, the pattern recognition receptors were all up-regulated. Among them, the expression of PGRP-S2 was highest in the transgenic line ov-AMP49 (Figure 7A) was significantly up-regulated after infection with *S. aureus* (Figure 7B). In addition, the immunomodulatory factors, *Tollip*, *Dorsal*, *Jun-D*, and *Lap2,* were up-regulated in ov-AMP49 and down-regulated in ov-AMP14. The reactive-oxygen-related genes *NOS1*-like, *SOD1*, and peroxidase were up-regulated in ov-AMP49 and down-regulated in ov-AMP14. *NOS1* and *SOD3* were up-regulated in the two transgenic strains, while only *NOS1*-like was significantly differentially expressed (Figure 7B).

The above results showed that the expression of most of the immune-related genes was up-regulated in ov-AMP49 and down-regulated in ov-AMP14. This suggests that we obtained a more immune transgenic line, namely, ov-AMP493.7. Validation of differentially expressed genes was performed using qRT-PCR.

**Figure 8:**
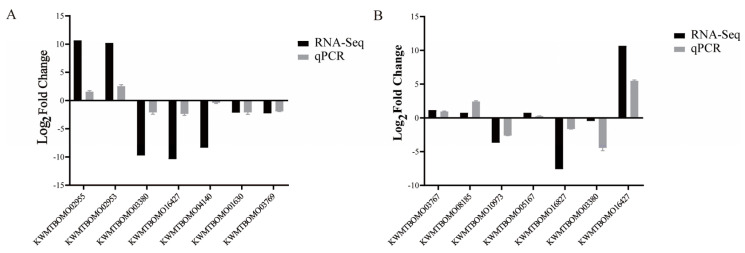
Verification of RNA-Seq results by qPCR. (**A**) qRT-PCR validation of partial genes in WT1 vs. ov-AMP14 samples. (**B**) qPCR validation of partial genes in WT2 vs. ov-AMP49 samples. Black bars represent RNA-seq data difference ploidy. The log2(FPKM-ov-AMPs/FPKM-WT) values were obtained by RNA-seq. Grey bars represent the qPCR difference ploidy. Three biological replicates were used to ensure statistical credibility. Student’s *t*-tests were used to detect differences between the transgenic and wild-type silkworms.

To verify the accuracy of the RNA-seq data, seven differentially expressed genes in the WT1 vs. ov-AMP14 and WT2 vs. ov-AMP49 groups were randomly selected for qRT-PCR analysis (Figure 8). The significance analysis of the qPCR data is shown in Supplementary Figure S1E. The expression patterns of 10 genes obtained via qPCR are consistent with the RNA sequencing data. These results indicate that the transcriptome sequencing data are accurate.

## 4. Discussion

The antimicrobial peptides of insects are one of the components of the innate immune system. Antimicrobial peptides are usually comprised of 12–50 amino acids [20] and exhibit antibacterial, antifungal, and antiviral activities [24]. In recent years, an increasing number of antimicrobial peptide genes have been cloned in different insects. The immune system of BSF has important research value because of the complex living environment of BSF. It has been reported that there are more antimicrobial peptide genes in BSF than in other insects [25,26,27].

In this study, two transgenic silkworm strains were obtained by initiating the expression of the antimicrobial peptide genes of BSF in silkworms using a midgut-specific promoter. To investigate the antimicrobial activity of the transgenic silkworm strains, we selected *S. aureus* and *E. coli* for antibacterial experiments on silkworms. The results showed that the ov-AMP49 strain had good antibacterial activity against *S. aureus*, while the ov-AMP14 strain had lower antibacterial activity than WT (Figure 2).

In 2009, researchers found that the antimicrobial peptide gene, *CG13551,* had antibacterial activity against Gram-positive bacteria in *Drosophila melanogaster* [28]. In contrast, the antimicrobial peptide gene, BSF AMP HiCG13551, a homologue of *Drosophila* CG13551, had a broader effect, with good antimicrobial activity against *E. coli* and *S. aureus* [7]. *Hidiptericin-1* belongs to the diptericin structural family of antimicrobial peptides. In 2001, a study found that diptericins had antibacterial activities against some Gram-negative bacteria, such as *E. coli*, while antibacterial activity against Gram-positive bacteria has not been reported [29]. In the present study, the antimicrobial effect of the ov-AMP49 strain against *S. aureus* was greater than that of the ov-AMP14 strain. This result may be due to the fact that the entry of exogenous genes reacted with the endogenous antimicrobial peptide of the silkworms, thus enhancing the resistance of the silkworms to Gram-positive bacteria. To further explore the roles of *HiCG13551* and *Hidiptericin-1* in immunization, the temporal expression profiles of the endogenous antimicrobial peptides *Cecropin* and *Gloverin,* after infection with *S. aureus,* were analyzed by qRT-PCR. It was reported that Gloverins are mainly active against *E. coli* [21], while Cecropins have a broad spectrum of activity against Gram-negative and Gram-positive bacteria [30]. Thus, after infection with *S. aureus,* the expression of *Gloverin* was up-regulated in ov-AMP49, which has a better antibacterial effect, and it was down-regulated in ov-AMP14. Notably, the *Cecropin* and *Gloverin* showed opposite trends in the two overexpression lines. It is hypothesized that the endogenous antimicrobial peptide *Cecropin* is required to respond to immune expression because ov-AMP14 is less antimicrobial, whereas ov-AMP49 is more antimicrobial at a level sufficient to respond to immune expression by the exogenous antimicrobial peptide *Hidiptericin-1*.

In this study, following the specific expression of antimicrobial peptide genes from BSF in the midgut of silkworms, the overexpression strains were infected with *S. aureus.* Wild-type silkworms served as the control group, and the midgut tissues were obtained and separated after 12 h for transcriptome sequencing. The transcriptome sequencing results revealed a total of 288 DEGs in the WT2 vs. ov-AMP49 group, while 476 DEGs were found in the WT1 vs. ov-AMP14 group (Figure S1). The WT1 vs. ov-AMP14 group was mainly enriched in the pancreatic secretion, starch and sucrose metabolism, protein digestion and absorption, and other pathways (Figure 6C). The WT2 vs. ov-AMP49 group was mainly enriched in the starch and sucrose metabolism, pantothenate and CoA biosynthesis, drug metabolism (other enzymes), biotin metabolism, platinum drug resistance, galactose metabolism, pancreatic secretion, and other pathways (Figure 6D). The functional annotation results of the differentially expressed genes showed that ov-AMP49 was more active in immune metabolic pathway responses and that the strain was more responsive to bacterial infection and external damage.

Six antimicrobial peptide genes are present in silkworms, which are produced by the Toll and IMD signaling pathways [31]. The innate immune system of insects includes a number of antimicrobial peptides, antiviral factors, and functions operating through a number of pattern recognition receptors (PRRs) [32], such as Toll-like receptors, the peptidoglycan recognition protein (PGRP) [33,34], and scavenger receptors (SRs) [35]. Such PRRs were all highly expressed in ov-AMP49, suggesting that *Hidiptericin-1* enhances innate immunity in silkworms (Figure 7B). Among the PRRs, PGRP-S5 was significantly up-regulated in the transgenic line ov-AMP49 after infection with *S. aureus* (log2(foldchange) = 2.42) and had a high expression level (Figure 7). This may be related to the role of PGRP-S5 as a regulator in the humoral immune system of the silkworm [3]. In this study, the expression of scavenger receptors and antimicrobial peptides was up-regulated in ov-AMP49 after infection with *S. aureus* (Figure 7B). This finding is consistent with the conclusion that scavenger receptors can enhance bacterial clearance and promote AMP production in vivo [36].

It has been shown that the midgut immune system of silkworms is capable of producing reactive oxygen species and antimicrobial peptides and that AMPs and ROS play synergistic roles in defense against microorganisms [37]. By analyzing endogenous antimicrobial peptides in silkworms, we found that *DefensinA* (log2(foldchange) = 2.72)*, Lebocin3* (log2(foldchange) = 1.75), and *Gloverin2* (log2(foldchange) = 2.59) were significantly up-regulated in the transgenic line ov-AMP49 (Figure 7B). *Gloverin* is found only in *Lepidoptera*, such as *B. mori* [21]. *Lebocin3* was reported to have a synergistic effect on *Cecropin D*, and their combination greatly increased the antimicrobial activity of *Cecropin D* in the silkworm [31]. The *BmDefensinA* of the silkworm is thought to be associated with insect Defensins [38]. Therefore, we suggest that, in the transgenic line ov-AMP49, the *Hidiptericin-1* gene may resist *S. aureus* infection by promoting the expression of *DefensinA*, *Lebocin3*, and *Gloverin2*.

Although the antibacterial peptides of the silkworm have prevented infection with some pathogenic bacteria, we are still committed to introducing exogenous genes into silkworm cells in order to obtain better antibacterial effects. In this way, the immunity of silkworms could be improved. However, there are some limitations of the present study. The silkworm strain used in this study was the experimental line D9L, and, in order to better apply the resistant silkworm strain in production, we would need to use the production silkworm strain for the experiments. In addition, we only used *E. coli* and *S. aureus* in the pathogenic bacteria infestation experiments, and the pathogenic bacteria species were not rich or diverse enough. In order to better apply the resistant silkworm strains in this study, we should also test the transgenic silkworm for its cocoon and silk qualities and other economic benefits. In this study, the effect of the overexpression of exogenous antimicrobial peptides on the immune system of silkworms was investigated by specifically expressing the antimicrobial peptide genes of BSF in the silkworm midgut. We also identified some pathways that may be related to the immune response of silkworms through transcriptome sequencing analysis in the hope of elucidating the mechanism of action of the antimicrobial peptides of BSF on the endogenous immune response in silkworms. In the future, we could screen other important antimicrobial peptide genes in BSF for overexpression in the silkworm. Notably, we could overexpress not only antimicrobial peptide genes, but also other immune pathway factors of BSF in silkworm to obtain more antimicrobial strains of silkworm. This study provides new insights for the study of silkworm disease resistance in the future.

## Figures and Tables

**Table 1:** Data on the numbers of embryos injected and larvae hatched.

Plasmid	Injection Embryos	Hatch	G0 Adults	Position G1
pbac-*HiCG13551*	600	180 (33.3%)	146 (24.3%)	15 (10.3%)
pbac-*Hidiptericin-1*	640	198 (30.9%)	164 (25.6%)	30 (18.3%)

## Data Availability

The data presented in this study are available upon request from the corresponding author.

## Supplementary Materials

The following supporting information can be downloaded at: https://www.mdpi.com/article/10.3390/insects14050443/s1, Figure S1: Cloning of target genes and expression levels in DEGs; Table S1: Primers used in this study.
