# Supplementary material for: Specific Expression of Antimicrobial Peptides from the Black Soldier Fly in the Midgut of Silkworms (Bombyx mori) Regulates Silkworm Immunity
Source: Insects. 2023 May 8;14(5):443. doi: 10.3390/insects14050443 (PMC10231060; doi:10.3390/insects14050443)
Supplement: Supplementary file 1 [file insects-14-00443-s001.zip › Figure S1.pdf]

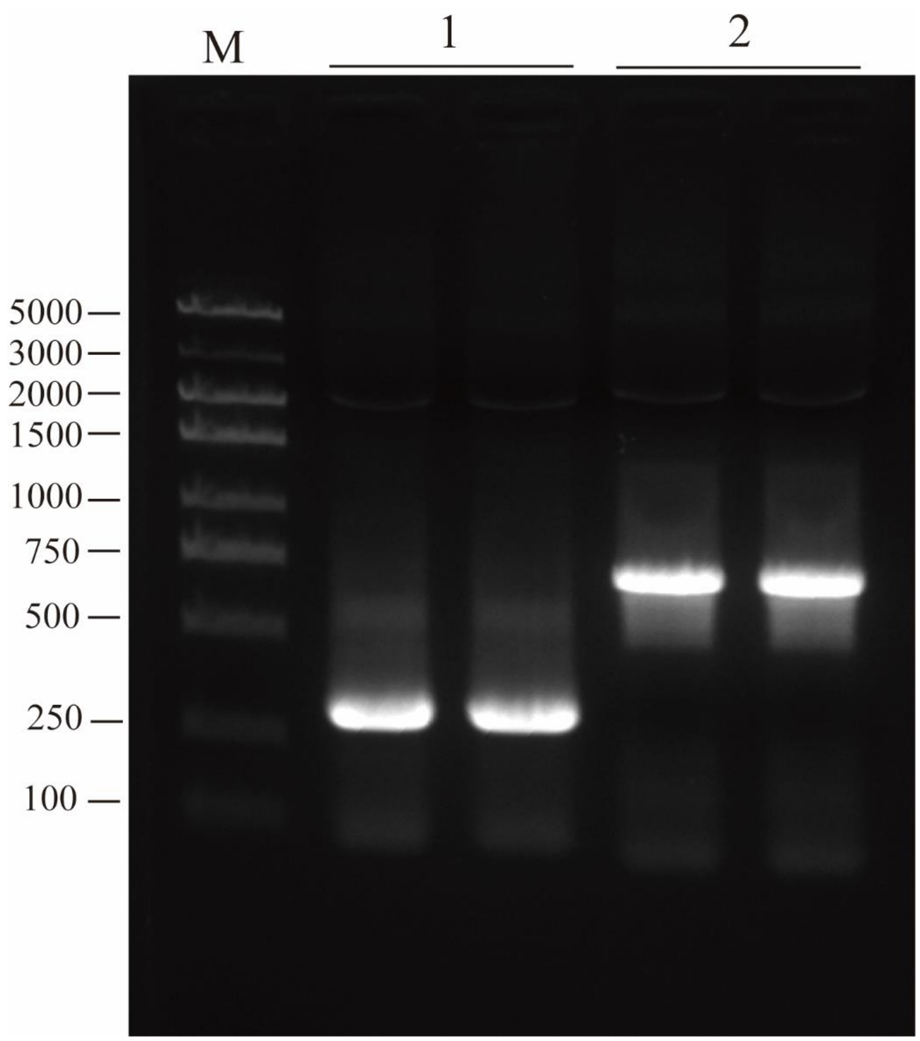

Figure S1-A

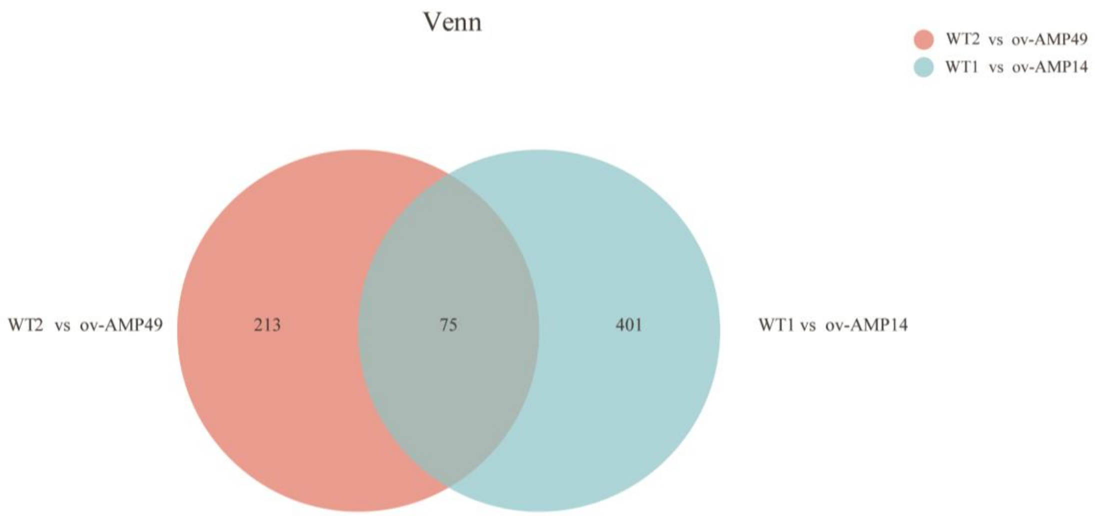

Figure S1-B

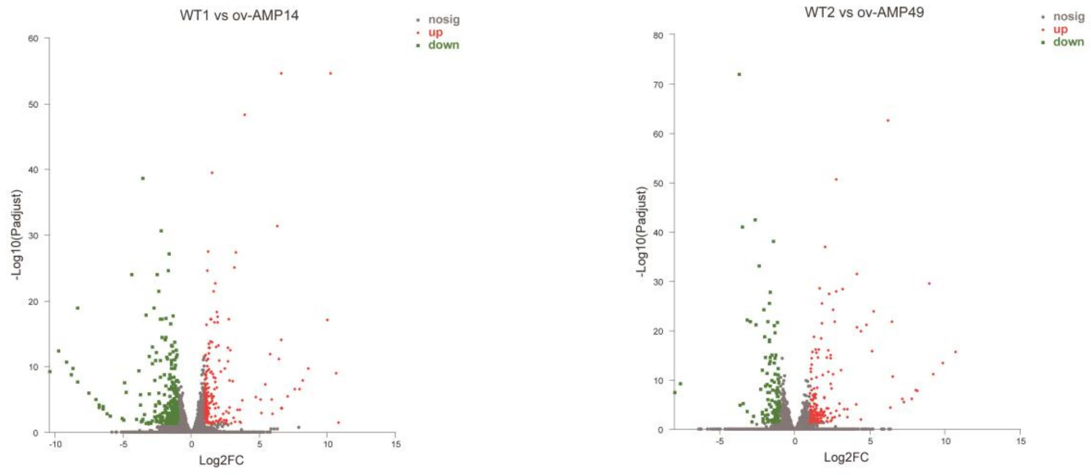

Figure S1 -C

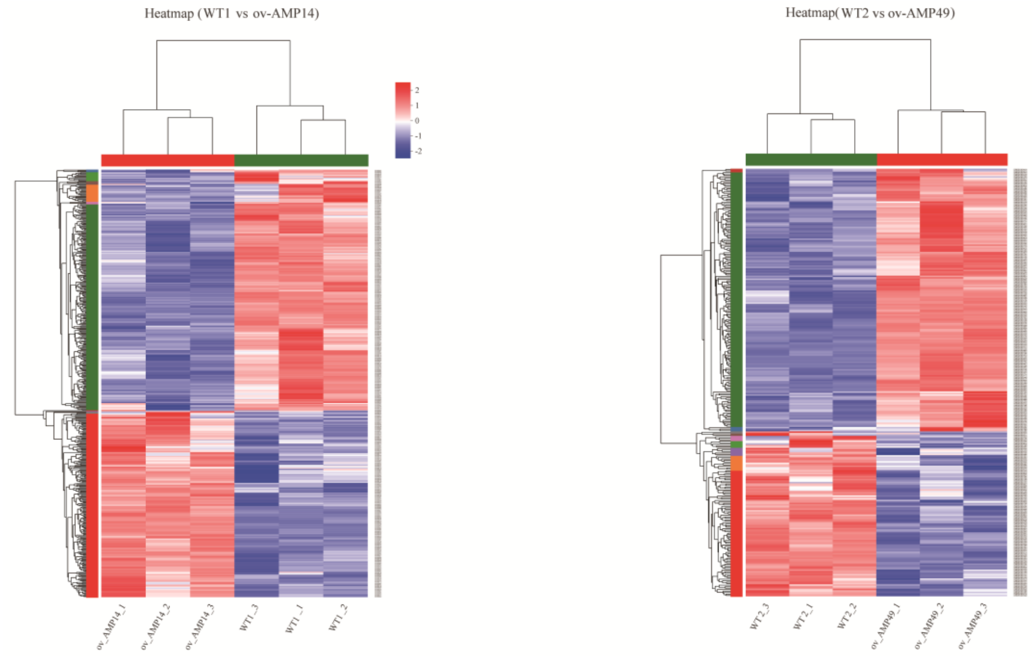

Figure S1-D

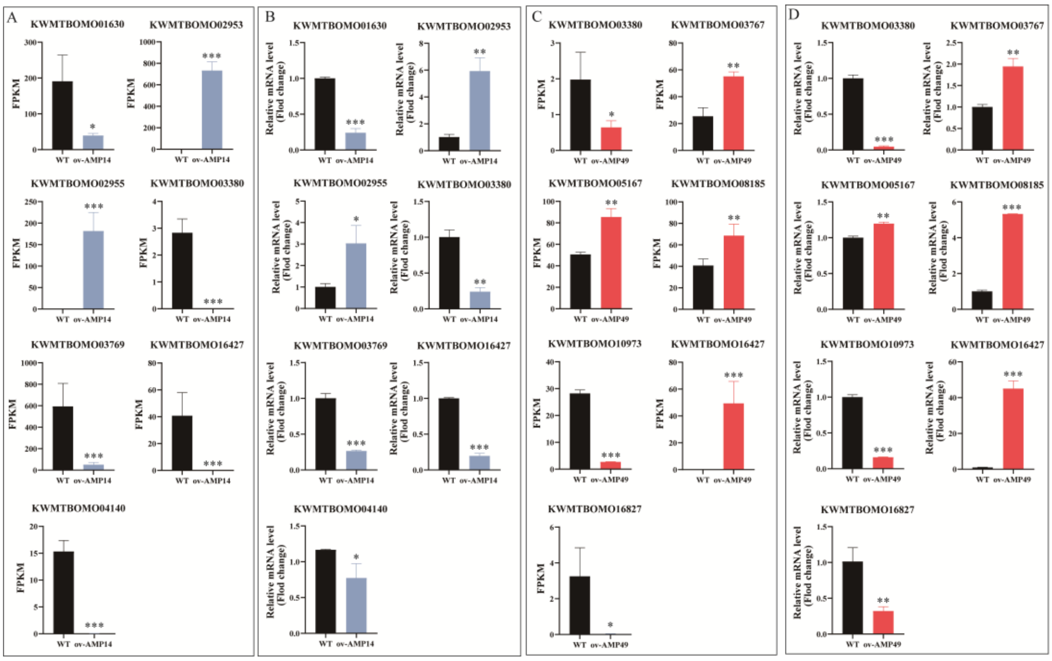

Figure S1-E

Figure S1. Cloning of target genes and expression levels in DEGs. (A) Cloning of two antimicrobial peptide genes from BSF. 1: *HiCG13551*. 2: *Hidiptericin-1*. (B) Venn analysis of two gene sets to identify common and unique genes between gene sets. (C) Expression difference volcano map of WT1 vs. ov-AMP14 group and WT2 vs. ov-AMP49 group. The horizontal coordinate is the number of fold of gene expression difference between two samples, and the vertical coordinate is the statistical test value of the difference in gene expression change, The larger the log10 (p-value) the more significant the expression difference. Each point in the graph represents a specific gene, red points indicate significantly up-regulated genes, green points indicate significantly down-regulated genes, and gray points are non-significantly different genes. (D) Expression difference cluster heatmap analysis of WT1 vs. ov-AMP14 group and WT2 vs. ov-AMP49 group. (E) A, C represent RNA-seq data difference ploidy; B, D represents qPCR difference ploidy. P-values were determined by the Student's T test. *P < 0.05, **P <0.01, ***P < 0.001, ns means no significant difference.
