# Supplementary material for: Specific Expression of Antimicrobial Peptides from the Black Soldier Fly in the Midgut of Silkworms (Bombyx mori) Regulates Silkworm Immunity
Source: Insects. 2023 May 8;14(5):443. doi: 10.3390/insects14050443 (PMC10231060; doi:10.3390/insects14050443)
Supplement: Supplementary file 1 [file insects-14-00443-s001.zip › Table S1.pdf]

Table S1 Primers used in this study.

Name	Primer sequence	purpose
HiOGS01149-F	CGGGATCCATG**GATTACAAGGATGACGACGATAAG**AGTTATAAGCTCTTAGCAC	Gene clone
HiOGS01149-R	ATAAGAATGCGGCCGCTTATCCACCTCCAAAGTTGG	
HiOGS03714-F	CGGGATCCATG**GATTACAAGGATGACGACGATAAG**AGCCAATTGGGAGACCTAG	
HiOGS03714-R	ATAAGAATGCGGCCGCCTATTTTTTCATCTTCTCAAGGAACTCC	
qHiOGS01149-F	TTACCAAGAAGTGCTCCAAAG	The relative mRNA levels of *HiOGS03714* and *HiOGS01149* were investigated by qPCR
qHiOGS01149-R	GTACACGACCATGAGAAGACG	
qHiOGS03714-F	CGGTGGTGGCTCCATTCGT	
qHiOGS03714-R	TTGCGGCCTCGTGTTCTGC	
SW-F	TTCGTACTGGCTCTTCTCGT	Reference gene
SW-R	CAAAGTTGATAGCAATTCCCT	
qCecropin-F	CTTCGTCTTCGCGTTGGT	qPCR analysis of the expression of endogenous antimicrobial peptides and immune-related genes in transgenic silkworms.
qCecropin-R	AAGGATTTCGCTTGCCCTAT	
qGloverin-F	GCACTTTGGGACAAAACGAT	
qGloverin-R	TGGCTTGTGCATTCTTGTTC	
qImd-F	CCGTATGCGAAAGGAA	
qImd-R	TTGGAAGGTAGAGAGG	
qKWMTBOMO03767-F	GGATTCTTCTCGGGAGTATGGG	Validation of differentially expressed genes using qPCR
qKWMTBOMO03767-R	AGCAGTGTGCAGCTGACAGGAC	
qKWMTBOMO08185-F	TTATTTTATTGTTCGCTTGC	
qKWMTBOMO08185-R	CACATTGCGTTGTAGATTTG	
qKWMTBOMO02953-F	GACGGCTGCCCTGCTTCCACATCA	
qKWMTBOMO02953-R	GCACACACGAACGCCACGCACAAA	
qKWMTBOMO03769-F	GCTGGATTATGATTTATGGT	
qKWMTBOMO03769-R	GTACGGATTGTTTTAGGGAC	
qKWMTBOMO02955-F	TATCGTAACATCACACATCACCTC	
qKWMTBOMO02955-R	ATATTGTACCAACGCACCAGACTC	
qKWMTBOMO01630-F	CGCCAACTCCACCTCCTACT	
qKWMTBOMO01630-R	CAATGCCATCACCATCACTG	
qKWMTBOMO05167-F	ACATCATACGAAGGCACCACAT	
qKWMTBOMO05167-R	CTGCTACTAAAGGACCACCCGA	
qKWMTBOMO04140-F	ATCTGCCTTTCGCGTACTTA	
qKWMTBOMO04140-R	TTGACACCGCTGTTCCCTTT	
qKWMTBOMO16427-F	TATTTCTTCGCCACGGTCC	
qKWMTBOMO16427-R	CCTCCCACTTGTTTATCCC	
qKWMTBOMO03380-F	AGCAGTTGATTGGAGAAGG	
qKWMTBOMO03380-R	ATGTTGCAGTGGAGTTGAG	
qKWMTBOMO16827-F	CCTGGAGGAAGAAGGCAAC	
qKWMTBOMO16827-R	CCAACCGCTTATTAAAGAT	
qKWMTBOMO10973-F	AGGGTTAGGTTTTTCTTTGT	

qKWMTBOMO10973-R	AATCCTGCTTGGTAGGGTACT

Note: The underline of primer represents the restriction site BamHI and NotI. Bold represents the Flag protein tag sequences.
